# A Brief Review on Multivalent Intercalation Batteries with Aqueous Electrolytes

**DOI:** 10.3390/nano6030041

**Published:** 2016-02-26

**Authors:** Ramesh K. Guduru, Juan C. Icaza

**Affiliations:** Department of Mechanical Engineering, Lamar University, Beaumont, TX 77710, USA; jicaza@lamar.edu

**Keywords:** aqueous batteries, multivalent ion batteries, rechargeable batteries, energy storage, intercalation batteries, specific capacity, aqueous electrolyte

## Abstract

Rapidly growing global demand for high energy density rechargeable batteries has driven the research toward developing new chemistries and battery systems beyond Li-ion batteries. Due to the advantages of delivering more than one electron and giving more charge capacity, the multivalent systems have gained considerable attention. At the same time, affordability, ease of fabrication and safety aspects have also directed researchers to focus on aqueous electrolyte based multivalent intercalation batteries. There have been a decent number of publications disclosing capabilities and challenges of several multivalent battery systems in aqueous electrolytes, and while considering an increasing interest in this area, here, we present a brief overview of their recent progress, including electrode chemistries, functionalities and challenges.

## 1. Introduction

There has been a growing requirement for high energy density charge storage devices for load leveling purposes in power grids, automotive vehicles, solar power generation, wind mills and many other large and small scale applications [1,2,3,4,5,6,7,8]. Currently, Li-ion batteries have been serving many of such applications ranging from small scale electronic gadgets to automotive vehicles. However, the long term concerns of high cost, limited reserves of lithium, and safety of Li-ion batteries have driven the researchers to search for alternative energy storage solutions. As a result, researchers have started to focus on other alkaline, alkaline earth and transition metal based rechargeable batteries [9,10,11,12,13,14,15,16,17]. For example, there is plenty of research done on Na-ion batteries [9,10,11,12] and these are inexpensive and somewhat less hazardous compared to the Li-ion batteries, but they are yet univalent and not adequate enough to meet the increasing energy density demands. On the other hand, multivalent cations, for example Mg^2+^, Ca^2+^, Zn^2+^, Ni^2+^, Al^3+^, *etc.*, have the ability to transfer more than one electron and thereby facilitate more energy storage than the univalent Li- and Na-ion batteries [16,18,19,20]. In addition, many of these multivalent ions are usually smaller than the univalent ions, and the volume changes associated with insertion of some of these ions are very similar to the volume changes observed in Li-ion battery electrodes, and it has also been supported by theoretical studies [21]. Considering the increasing demand for high energy density batteries and improved safety, in this article we mainly focus on reviewing the aqueous electrolyte based multivalent intercalation batteries.

## 2. Overview on Multivalent Intercalation Batteries

The multivalent cation chemistries are principally advantageous for their energy density and improved safety. In fact, many of these multivalent ions are light enough to deliver high energy densities compared to the Li-ion batteries. Table 1 shows a comparison of different multivalent metal anodes in terms of their standard electrode potentials, and specific and volumetric capacities with reference to Li and Na anodes. However, in practice, the development of multivalent battery systems is often hindered by lack of suitable cathode chemistries that show a good reversibility of ion intercalation.

There were some initial efforts in the 1990s by Gregory *et al.* [22] and Novak *et al.* [23,24,25] on non-aqueous multivalent Mg-ion batteries. Gregory *et al.* [22] showed a successful chemical and electrochemical insertion of Mg^2+^ into various transition metal oxides and sulfides. Novak and coworkers’ [23] work showed some important directions on the intercalation of water solvated Mg^2+^ into V_2_O_5_ and MoO_3_ electrodes using aprotic electrolytes with small amounts of water molecules. These studies revealed a screening effect of water on Mg^2+^ during the intercalation process. Later, many researchers investigated the intercalation behavior of polyvalent ions in various electrode materials, including aerogels [23,26,27,28,29,30,31,32,33,34,35,36,37,38,39,40]. In 1998, for the first time, Le *et al.* [29] investigated the intercalation of Al^3+^ and Mg^2+^ in V_2_O_5_ aerogels, and they also reported the advantage of water molecules in the V_2_O_5_ aerogel structures providing steric hindrance effect on intercalating Mg^2+^ and thereby aiding in a good reversibility of multivalent ion insertion into V_2_O_5_ aerogels. Following this work, many researchers studied the intercalation of Mg^2+^ into the V_2_O_5_ aerogels and their composites [29,30,31,32,33]. In 2000, it was Aurbach’s work [41] that further optimistically directed the feasibility of multivalent intercalation based Mg-ion battery technology using ether electrolytes. Unlike Li and Na, Mg can be easily used as an anode in Mg-ion batteries because of less reactiveness in the ambient atmosphere, and absence of dendritic growth issues during electrodeposition. In addition, Mg is very abundant, inexpensive and offers low standard electrode potentials along with a large theoretical capacity, see Table 1. Similarly, the other multivalent metals (e.g., Al, Ca, Y, Ni, and Zn *etc.*) also offer several advantages in terms of ease of handling, abundance, low cost, high storage capacity and energy density (see Table 1), and many of these could be used as anodes in aqueous electrolytes, but they are all restricted by limited choice of cathode and electrolyte materials [15,16,42,43].

The electrolyte challenges are magnified for multivalent batteries, and their choice is very critical in terms of high ion mobility and facile (de)solvation of the ions at the electrode interfaces [18,44]. The desolvation energy can be a measure of the energy required to detach the cation from its solvent solvation shell. For example, the passive layer of solid-electrolyte interface formed between the electrolyte and the electrodes reduces the extent of diffusion of alkaline earth metal ions, unlike in Li-ion batteries where only electrons are hindered and not the Li-ions [18]. As explained above, the high charge density of Mg-ion contributes to a larger desolvation energy and thereby impede its diffusion into the electrode at the interface. Thus, the nature and interaction of multivalent cation with the solvents and counter anions are very critical in multivalent electrolytes. The larger size of Ca^2+^ with reduced charge density could help in easy diffusion of Ca^2+^ into the electrodes, but lack of suitable electrolytes, less capacity and reversibility issues make it less attractive. The multivalent salts are also not very soluble in well studied organic solvents, and some of them could be very corrosive [18]. Thus, the advancement of multivalent batteries is very dependent on the discovery of both high voltage cathode materials and electrolytes, the electrode-electrolyte interface reactions, and the diffusion of multivalent ions.

The common approaches to mitigating the interface and solid state diffusion issues of multivalent ions include nano sizing of the electrode materials to shorten the diffusion distances, application of electrode materials with large tunnel structures or interlayer spacing, and partial shielding of the multivalent ions with polar molecules (e.g., water). The electrodes with larger spacing will help minimize the steric hindrance effects [19]. The nanostructures of electrodes will also reduce the impact of structural distortions during intercalation and de-intercalation of the multivalent ions. However, finding a suitable organic solvent with more solubility of multivalent salts, and achieving a good reversibility are yet challenging in many cases. In addition, many of these organic solvents are very sensitive to oxygen and moisture, and are very flammable and toxic as well. The safety issues are a major concern with the organic solvents as overcharging and heating could lead to their evaporation and result in explosion of the batteries. There are many accidents reported in the recent past related to these phenomena [45,46,47]. The best way to deal with such problems is replacing the organic electrolyte with an aqueous electrolyte, which also provides the advantage of being abundant, low cost, and environmentally benign [48,49]. In 1994, Dahn *et al.* [50] used aqueous electrolyte for Li-ion batteries. Following this paper, aqueous electrolytes were used in many other batteries [16,17,18,19,20,33,43,51,52,53,54,55,56,57,58,59,60,61,62,63,64,65,66,67,68,69,70,71,72,73,74,75,76,77]. In 2012, a rechargeable multivalent Al-ion battery with aqueous electrolyte was proposed as a promising candidate for high energy storage to compete with the Li-ion batteries [51]. However, it should be noted that the intercalation reactions are usually very complicated in aqueous electrolytes. There could be different side reactions, such as co-intercalation of proton into the electrode materials along with the intercalation of multivalent ions, reaction of electrode materials with water or oxygen, hydrogen/oxygen evolution reactions, and the dissolution of electrode materials in water depending on the pH. The proton co-intercalation could happen in the positive electrode materials, and it usually depends on the crystal structure of the electrodes and pH of the electrolyte. For example, spinel Li_1−*x*_Mn_2_O_4_ and olivine Li_1−*x*_FePO_4_ do not encounter such proton insertion, whereas delithiated layered Li_1−*x*_CoO_2_ and Li_1−*x*_Ni_1/3_Mn_1/3_Co_1/3_O_2_ show a significant concentration of protons in the lattice during deep lithium extraction with a low pH or more acidic electrolyte [68,69]. Deep extraction of Li results in lower oxidation state of transition metals, which is possibly compensated with exchange of protons from the reaction medium, *i.e.*, electrolyte. Therefore, control of pH and corresponding voltage window selection are very critical [70]. Importantly, the voltage window of the batteries should also be well controlled as a function of pH to avoid H_2_/O_2_ evolution reactions, and this in turn could influence the capacity of the electrode materials. For example, the extent of Li-ion extraction is affected by pH of the electrolyte and corresponding voltage window in the case of LiMn_2_O_4_, whereas it has less effect in the case of LiFePO_4_ in the range of 7–14 [71]. Another important fact is the interaction of the electrode with water, which should not be soluble in water. For example, electrode materials of VO_2_, LiV_3_O_8_ and LiV_2_O_5_ with nanostructures or large surface area are not a good choice in aqueous electrolytes [71]. It should also be noted that some of the electrode materials decompose in strong alkaline solutions and it needs to be prevented through the application of appropriate coating materials, for example carbon based coatings [72].

The current research status of aqueous multivalent batteries indicates positive future prospects along with a huge requirement for further discoveries and developments of new materials and chemistries in order to extend their implementation to practical uses. Thus realizing their future potentials, here we provide a brief overview on the research progress of aqueous multivalent intercalation batteries and the challenges encountered.

## 3. Literature Review on Multivalent Aqueous Batteries

### 3.1. Al–Ion Batteries

Although Al^3+^ is smaller than Li^+^ in the unsolvated state, its strongest electrostatic bonding nature with the host electrode materials, similar to many other di- and tri-valent ions, usually results in slower diffusion kinetics within the electrodes. Despite this, it has been found to successfully intercalate and deintercalate in certain compounds exhibiting good electrochemical characteristics for battery applications. Following are the details of compounds explored for Al-ion battery cathode applications with aqueous electrolytes.

- ***TiO_2_***

In 2012, for the first time, Liu *et al.* [51] demonstrated storage of Al^3+^ in anatase TiO_2_ nanotube array (NTA) structures in aqueous AlCl_3_ electrolyte. The insertion of Al^3+^ into NTA resulted in the reduction of Ti^4+^ to Ti^3+^/Ti^2+^, but the extraction process oxidized only Ti^3+^ back into Ti^4+^ with an irreversibility in the oxidation of Ti^2+^ [56]. Thus, the fundamental reaction that helped in storing Al^3+^ in TiO_2_ was the redox reaction pair of Ti^4+^/Ti^3+^. In their studies, the nanostructures of TiO_2_ with a large surface area on a Ti substrate facilitated a good electrode/electrolyte contact and thereby a fast diffusion pathway for Al^3+^ into the anatase tubes. A maximum discharge capacity of 75 mAh/g was obtained. The cyclic voltammetry (CV) studies showed a clear redox couple at −1.26 and −0.84 V (*vs*. SCE) for Al^3+^ insertion and extraction, respectively. In these electrodes, a high over potential of −1.43 V (*vs*. SCE) for hydrogen evolution was attributed to a strong solvation of Al^3+^ in the aqueous electrolytes. It was also determined that the insertion and extraction of Li^+^ and Mg^2+^ was weaker for the same aqueous electrolyte, which supported the advantage of smaller Al^3+^ size compared to the Mg^2+^ and Li^+^ for a stronger reversibility of Al^3+^ insertion and extraction. These studies also indicated a predominant solid phase diffusion reaction for Al^3+^ insertion in the anatase TiO_2_.

Similarly, Liu *et al.* [54] also reported a promising performance of anatase TiO_2_ NTA in another study. They evaluated the performance of NTA of TiO_2_ in a series of electrolytes 0.50 M Al_2_(SO_4_)_3_, 1.00 M AlCl_3_, and two groups of mixed solutions (a) 0.25 M Al_2_(SO_4_)_3_ with different concentrations of NaCl solution; (b) 1.50 M NaCl mixed with different concentrations of Al_2_(SO_4_)_3_ solution, in order to understand the effect of chloride (Cl^−^) ions in the insertion process of Al^3+^ into the host structures. They obtained a discharge capacity of 74.8 mAh/g at a current density of 4 mA/cm^2^ without altering the morphology and crystal structure of NTA. This superior performance was attributed to the small radius of Al^3+^ (53.5 pm *vs.* 76 pm of Li^+^) and presence of Cl^−^ in the electrolyte. The CV studies of NTA showed no redox couple in Al_2_(SO_4_)_3_ electrolyte, whereas the redox peaks were around −1.2 and −1.0 V (*vs*. Ag/AgCl) in AlCl_3_ electrolyte. Further investigations in a series of electrolytes with varying concentrations of NaCl in Al_2_(SO_4_)_3_ showed an increase in the peak currents of redox couples and thereby supported the positive contribution of Cl^−^ in the insertion and extraction process of Al^3+^. These authors inferred a possible assistance of Cl^−^ in the insertion of Al^3+^ into the interstitial sites of TiO_2_ lattice, however the exact role was not presented and needed further investigation.

Following these articles, He *et al.* [53] also reported a similar investigation on nano leave structures of anatase TiO_2_ for Al-ion battery electrode applications. In their studies, the electrochemical performance of TiO_2_ nano leaves were investigated in Al(NO_3_)_3_ aqueous solution. The TiO_2_ nano leaves were found to facilitate Al^3+^ diffusion and electronic transport very easily without much of volumetric changes. As a result, a high aluminum storage with a capacity of 278 mAh/g at a current density of 0.05 A/g was reported. They also showed excellent cycling stability with a capacity of 141.3 mAh/g at a current density of 2.0 A/g, and such superior characteristics were ascribed to the ordered nanoparticles in the electrodes with improved intercalation and interfacial storage capacities. The lower polarization effects with a smaller potential drops or smaller hysteresis between the charge and discharge voltages indicate very low over-potentials for either insertion or extraction of Al^3+^ into the electrodes, and thereby helped in achieving enhanced performance. Similar behavior was also observed by Cui *et al.* [43] during insertion of Al^3+^ and many other trivalent ions into open frame structured Copper Hexacyanoferrate (CuHCF), where insertion of trivalent ions was associated with lower hysteresis between the charge-discharge curves. He *et al.* [53] has also confirmed interfacial storage of Al^3+^ in the nanoparticles of electrodes of anatase TiO_2_, unlike the usual battery electrodes which tend to form resistive solid electrolyte interfaces and result in a reduced capacity. Their observations were also supported without decomposition of the electrolyte with a high surface area of nano leave morphology providing the interfacial storage for Al^3+^.

- ***Copper Hexacyanoferrate (CuHCF)***

**Figure 1:**
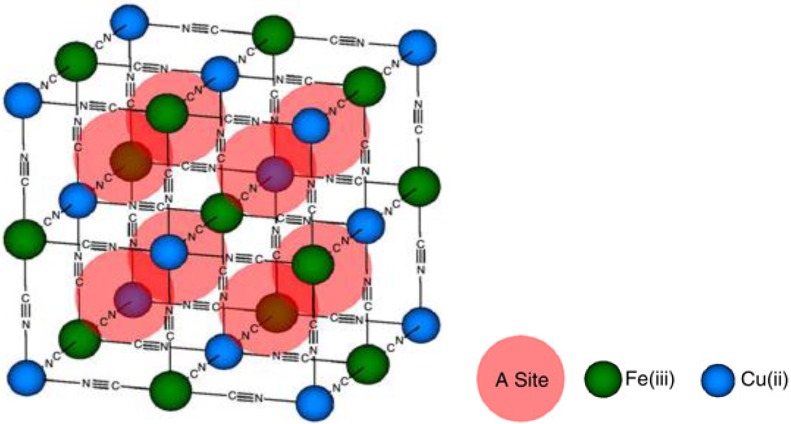
The crystal structure of Copper Hexacyanoferrate (CuHCF) (Reproduced with permission of [57]. Copyright Nature Publishing Group, 2015).

The electrode materials, such as CuHCF, with Prussian Blue analogues (PBA) structures showed a very good potential for multivalent ion insertion/de-insertion characteristics. The PBAs with a generic formula of A*_x_*PR(CN)_6_ contain P and R ions, which are separated and bonded by CN ligands forming a face-centered cubic structure with open framework as shown in Figure 1, and the large interstitial A sites are where the insertion of guest ions is possible. PBA’s electrochemical properties have been extensively studied for hydrogen storage, battery electrodes and electrochromic applications [57,58,78,79,80,81,82]. It has been recently demonstrated that copper and nickel hexacyanoferrates with PBA structure can electrochemically insert ions in aqueous solutions [57,58]. The research conducted by Wessells *et al.* [57,58], Cui *et al.* [43] and Steingart *et al.* [63] indicate that the capacity of CuHCF is insensitive to the ionic valency of the intercalating species, provided the concentration of the intercalating ions in the electrolyte is more than the moles of active material, and this is so because the number of ions inserted in the CuHCF depends on balancing of charge with the number of available Fe^2+^ in the crystal structure, and thereby limit the cell capacity.

In 2014, Liu *et al.* [52] investigated the storage of trivalent Al-ions in CuHCF for the cathode applications using a 0.5 M Al_2_(SO_4_)_3_ aqueous electrolyte. The theoretical specific capacity of CuHCF was predicted to be around 58.9 mAh/g based on the molecular weight of the compound, however the electrochemical measurements by Liu *et al.* [52] showed a specific capacity around 62.9 mAh/g at a current density of 50 mA/g, and it decreased to 46.9 mAh/g at a current density of 400 mA/g with a retention of 74.6% of the starting capacity. This superior performance was attributed to the shielding effect of zeolitic water on the motion of Al^3+^ in the host crystal structure with steric hindrance effects. The cyclic charge–discharge tests also showed a decent capacity up to 22.5 mAh/g after 1000 cycles. These studies clearly presented the CuHCF as another potential cathode material for Al-ion batteries [52].

In another study by Cui *et al.* [43], CuHCF was proved to be capable of reversibly intercalating many trivalent and divalent ions in the aqueous electrolytes. Their galvanostatic charge-discharge tests showed a lower voltage hysteresis for insertion of trivalent ions compared to the divalent ions and they attributed it to high level hydration of trivalent ions, which was also confirmed through structural analysis via Rietveld refinement. Interestingly, they also observed a negligible impact of insertion of ions with varying oxidation states into the CuHCF because of a high degree of screening effect by water molecules in the hydration shell or in the crystal structures minimizing the electrostatic interaction between the insertion ion and the host ions of CuHCF. In addition, the intercalation of multivalent ions was observed to show multiple cathodic and anodic peaks at distinct voltages, representing possible insertion/de-insertion of ions into/from multiple distinct crystallographic sites, suggesting the role of ferricyanide vacancies, which was also confirmed by Reitvald refinement. These vacancies are expected to help in providing diffusion pathways facilitating the migration of multivalent ions as well as charge redistribution to stabilize the crystal structure of CuHCF or PBA phases. Overall, this research suggested a critical role of vacancies and screening effect of water molecules on the reversible insertion of multivalent ions, which make PBA materials suitable electrode materials for multivalent batteries with their remarkable electrochemical characteristics.

### 3.2. Zn–Ion Batteries

Among several multivalent batteries, Zn-ion intercalation batteries have gained a considerable attention to replace conventional Li-ion batteries for large scale applications. The electrodes used for Zn-ion batteries are environmentally very benign and they can be manufactured very economically. The electrolytes are also very simple in chemistry [17,55]. There have been growing efforts toward developing high energy density intercalation batteries based on Zn-ion chemistries, and so far multiple materials have been investigated for cathode applications, which are discussed below along with the details of mechanisms and challenges.

- **α*-MnO_2_***

**Figure 2:**
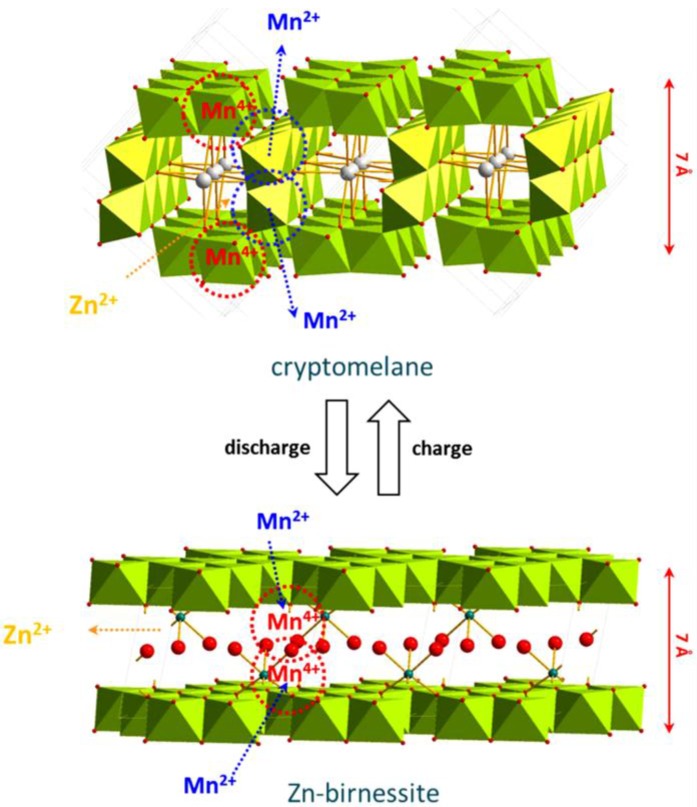
The reversible phase transition of α-MnO_2_ into triclinic Zn-birnessite upon intercalation of Zn ion into α-MnO_2_ during discharge/charge processes. (Reproduced with permission of [17]. Copyright Nature Publishing Group, 2015).

Although some types of MnO_2_ are currently being used in commercial alkaline batteries, they are gaining increased attention as possible electrodes for Zn-ion intercalation batteries also. The crystal structure of α-MnO_2_ has a tunnel structure as shown in Figure 2 (cryptomelane), and insertion of alkali, alkaline earth and post transition metals into these tunnel structures has been a long time research focus. Some of the important qualities of α-MnO_2_ in intercalation batteries include large discharge capacities with a practical discharge potential of 1.3 V (*vs.* Zn/Zn^2+^) at a moderate currents, and an energy density of 225 Wh/kg in 1 M aqueous ZnSO_4_ electrolyte. However, these intercalation batteries suffer from capacity fading in long term cycling with poor performance at high current rates [17,55].

Xu *et al.* [55] reported a reversible intercalation of Zn^2+^ into the tunnel structures of α-MnO_2_ resulting in a specific capacity of 210 mAh/g, which is relatively very large compared to the capacity delivered by Zn/MnO_2_ alkaline batteries (125 mAh/g). At a slow current rate of C/20, they exhibited a capacity retention up to 30 cycles with a very high columbic efficiency. Some of the most appealing points of these batteries include use of non-toxic, non-corrosive, and low-cost mild electrolytes with abundant raw materials [55]. In another study, Lee *et al.* [17] investigated the mechanism for intercalation of Zn^2+^ into α-MnO_2_ nano rods in order to understand the decay in their intercalation performance. This work discovered a reversible phase transition of α-MnO_2_ structures into a layered triclinic Zn-birnessite, as shown in Figure 2. The interaction of Zn^2+^ with the tunnel structures of α-MnO_2_ was found to be quite strong because of large electrostatic forces compared to Li^+^ and Na^+^, and it has been observed to change the oxidation state of Mn upon intercalating into the tunnel structures. Further investigation through X-ray near edge absorption structure (XANES) studies revealed the dissolution of unstable Mn^3+^ from the walls of the tunnels into the electrolyte causing a phase transition from the tunneled to layered structure. Interestingly, the deposition of Mn on Zn anode was not observed, and rather the Mn ions were observed to reversibly insert back into the layer structures of Zn-birnessite during the charging process. With increased number of cycles, electrodes turned into amorphous phase due to excessive stresses generated during the phase transitions of α-MnO_2_ and Zn-birnessite, and it caused the capacity to fade. However, Lee *et al.* [17] did not propose any alternatives to improve the cycling behavior of α-MnO_2_.

Recently in 2015, Xu *et al.* [59] also worked on developing Zn-ion intercalation batteries while developing Ni-ion batteries. They conducted both theoretical modeling and experimental work based on supercapacitor’s research with an inspiration of increasing capacitance and charge rate capability of batteries while increasing the valence of ions in the electrolyte. Their theoretical calculations predicted a thermodynamically favorable insertion of Zn^2+^ into different lattice sites of the tunnel structure of α-MnO_2_ with a binding energy ranging from −1.05 to −2.09 eV. Their experimental work also supported the theoretical modeling studies and yielded a specific capacity around 220 mAh/g. However, it should be noted that they used a 20 wt % of graphene as an active ingredient along with 70 wt % of α-MnO_2_ in their electrodes, unlike the earlier work on α-MnO_2_ in references [17] and [55], and it helped not only in obtaining a slightly higher capacity but also a higher energy density of 320 Wh/kg.

- **λ*–MnO_2_***

In an interesting study by Yuan *et al.* [20], λ-MnO_2_ was synthesized by removing Li from LiMn_2_O_4_ via selective acid leaching process. They investigated the electrochemical performance in an aqueous ZnSO_4_ electrolyte for Zn-ion intercalation battery electrode applications. The oxidation and reduction reactions were determined to correspond to the transformation of Mn^3+^/Mn^4+^, and Mn^4+^/Mn^3+^, respectively, and they showed an initial discharge capacity of 442.6 mAh/g with a coulombic efficiency of nearly 100%, however, at a current density of 408 mA/g, the capacity dropped to 33.8 mAh/g [20].

- ***Todorokite***

**Figure 3:**
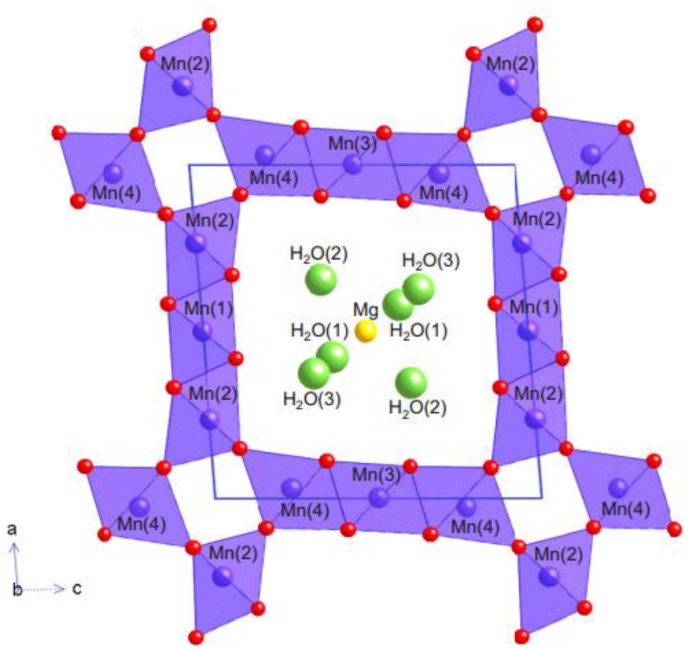
The crystal structure of Todorokite MnO_2_ mineral with a 3 × 3 tunnel structure and zeolitic/crystal water inside the tunnels (Reproduced with permission from [83]. Copyright Mineralogical Scoiety of America, 2015).

Todorokite is a type of manganese oxide (MnO_2_) mineral with a chemical composition of Mg_1.8_Mn_6_O_12_ 4.8 H_2_O consisting 1-dimensional 3 × 3 tunnel structure along the b-axis, see Figure 3 [83].

Many cations can intercalate into this large tunnel and stabilize the whole structure, including alkali, alkaline-earth, transition-metal ions, post-transition metals and water molecules [19]. The tunnel structures of Todorokite are bigger than the tunnels of α-MnO_2_ and facilitate easy diffusion of Zn^2+^. In addition, the crystallographic water inside the tunnels is expected to provide a partial shielding effect on Zn^2+^ from the walls of the tunnels, and thereby reduce the electrostatic interactions and bonding of Zn^2+^. In 2013, Lee *et al.* [19] studied the intercalation of Zn^2+^ into a synthetic Todorokite using a 1 M ZnSO_4_ aqueous electrolyte with Zn metal anode. The electrochemical characterization showed a specific capacity close to the theoretical value of 99 mAh/g with a cycle stability up to 50 cycles. However, the capacity of Todorokite was less than half of the capacity of α-MnO_2_ (210 mAh/g) [55], and it was attributed to the preexistence of Mg^2+^ in the tunnel structures. The pre-intercalated Mg^2+^ is expected to reduce the average oxidation state (AOS) of Mn to 3.4 compared 3.67 in α-MnO_2_. Therefore, a lower AOS of Mn leads to a lower amount of Zn^2+^ intercalation into Todorokite compared to the α-MnO_2_ and hence the lower specific capacity. In the second cycle of discharge, the same Todorokite was observed to show a higher capacity of 108 mAh/g because of partial removal of pre-intercalated Mg^2+^. Although, the discharge capacity of Todorokite was lower than α-MnO_2_, the capacity retention at high current rates was better, and it was credited to the large 3 × 3 tunnel structures and partial shielding effect of crystallographic water in Todorokite tunnels [53].

- ***Zinc Hexacyanoferrate (ZnHCF)***

Leyuan Zhang *et al.* [60] investigated the electrochemical insertion characteristics of Zn^2+^ into rhombohedral structured ZnHCF in aqueous electrolytes along with other monovalent cations (K^+^ and Na^+^). The electrochemical characterization with a metallic Zn anode and aqueous ZnSO_4_ electrolyte in a half cell configuration yielded a capacity of 64.4 mAh/g (at 5C) and 73.7 mAh/g (at 1C). In contrast, a full cell configuration of Zn anode against ZnHCF intercalated at a voltage of 1.7 V (*vs.* Zn/Zn^2+^) exhibiting a capacity of 65.4 mAh/g, and this intercalation voltage was reported to be one of the highest voltages for aqueous Zn-ion batteries. The specific energy density of these full cells was determined to be 100 Wh/kg based on the total mass of the active electrode materials. The cyclability studies also yielded a good performance with a retention up to 76% of initial capacity after 100 cycles, and this research certainly indicated the advantage of ZnHCF electrodes with high voltage for Zn-ion battery applications. However, authors did not provide the details of pH of the electrolyte and possible reasons for high voltage of the full cell configuration.

- ***Copper Hexacyanoferrate (CuHCF)***

In 2013, Jia *et al.* [61] studied the electrochemical intercalation of Zn^2+^ into CuHCF in aqueous ZnSO_4_ electrolyte. They demonstrated a successful reversibility of intercalation of Zn^2+^ with a specific capacity around 56 mAh/g. Most importantly, their CV studies showed a linear relationship between the peak current densities and the square root of scan rates employed indicating a solid-phase diffusion process. Consequently, it was determined that the diffusion of Zn^2+^ into CuHCF was harder than the extraction process using the scan rate and current density relationships in anodic and cathodic scans, which correspondingly exhibited a two stage discharge process at two different voltages compared to a single stage charging process in the galvanostatic charge-discharge tests. The authors attributed the successful insertion and extraction of Zn^2+^ into CuHCF to the small radius steric effect of Zn^2+^. In 2014, Trócoli and La Mantia [62] are the first to report a high voltage aqueous electrolyte battery using CuHCF. They conducted research on developing a Zn-ion battery with a 1.73 V using metallic Zn anode in a ZnSO_4_ electrolyte in three electrode configuration (Ag/AgCl–reference). During their electrochemical characterization, they made sure to maintain the pH of electrolyte around 6 in order to avoid passivation of Zn anode. The galvanostatic cycles for the positive (CuHCF) and negative (Zn) electrodes (at 1C) exhibited a cell capacity of 60 mAh/g. They observed a reduction potential for CuHCF around 0.9 V (*vs*. Ag/AgCl) and the oxidation potential for Zn anode around −0.83 V (*vs*. Ag/AgCl) and thereby they obtained a full potential of 1.73 V (*vs.* Zn/Zn^2+^) for the cell between CuHCF and Zn. However, testing of full cells was never performed by these authors. The cyclability data showed a very good capacity retention up to 96.3% of initial capacity after 100 cycles. They claim that the specific energy (56.3 Wh/kg) and specific power (794.3 W/kg) of their cells were close to the performance of Li_4_Ti_5_O_12_ and LiFePO_4_ electrodes in organic electrolytes at 10C.

In another recent study by Cui *et al.* [43] the divalent ion insertion, including Zn^2+^, showed reversibility in CuHCF but their capacity faded over a long period of cycling. The intercalation of Zn^2+^ exhibited a starting specific capacity around 40 mAh/g and it reduced to 20 mAh/g (at 5C), and this was attributed to possible swapping of Zn^2+^ ions with the metal ions of CuHCF frame work, but it was very inconclusive. The CV studies showed multiple cathodic and anodic peaks suggesting the assistance of ferricyanide vacancies during the diffusion process, which was also confirmed through the crystal structure analysis using Reitvald refinement. In addition, presence of water molecules in the CuHCF frame work were also reported to assist in the diffusion of multivalent ions in CuHCF crystal through shielding or steric hindrance effect. However, the reasons for decay in capacity with insertion of Zn^2+^ was not examined in detail by Cui *et al.* [43]. Steingart *et al.* [63] investigated this capacity decay phenomenon while comparing the electrochemical characteristics obtained using an aqueous Na_2_SO_4_ electrolyte. In this work CuHCF was cycled against hyper-dendritic zinc (HD Zn) anode in an acidic Na_2_SO_4_ solution. With Na^+^ as the primary ion for charge transport, a capacity retention of 90% (83)% after 300 (500) cycles at 5C was achieved, which was five times larger than any other PBA-Zn systems reported above. The maximum specific capacity of CuHCF was found to be 60 mAh/g irrespective of insertion of Zn^2+^ or Na^+^. But its rapid decay in capacity against Zn^2+^ suggested a possible irreversible damage of the crystal structure of CuHCF during the rapid removal and insertion of Zn^2+^. X-ray photo spectroscopy results showed Zn 2p peaks both in charged and discharged electrodes of CuHCF tested against Zn anode indicating accumulation of Zn in the CuHCF crystal, which corroborated with the authors assertion. They also observed a decrease in capacity with increasing storage time due to diffusion of Zn into the CuHCF crystal structure. Their investigation of electrochemical characteristics using HD Zn provided insights into the kinetics of Zn^2+^ diffusion. The large surface area of HD Zn lowers the over-potential and helps faster kinetics during the charge/discharge steps. They also showed more retention of capacity with HD Zn compared to the regular planar sheet electrode of Zn, and it could be because of the initial dendritic structure that takes time to densify before it starts to behave like a planar Zn sheet and also helps in reduction of unwanted volume expansion and formation of sharp structures avoiding soft shorts.

### 3.3. Ni-Ion Batteries

- ***α-MnO_2_***

As earlier explained in Zn-ion batteries, the research on Ni-ion batteries was pursued by Xu *et al.* [59] in 2015 with an inspiration from supercapacitor research. They argue that the insertion of multivalent ions into certain types of host materials with large tunnel structures is easier both thermodynamically and kinetically, which is against the usual notion of slower kinetics for multivalent insertion into the host materials compared to the univalent ions. Their theoretical modeling and experimental research focused on using α-MnO_2_ as a cathode for Ni-ion batteries. The modeling studies showed a favorable insertion of Ni^2+^ into different lattice sites of tunnel structure in α-MnO_2_ with binding energies ranging from −2.92 to −5.54 eV. The experimental investigations were carried out using an aqueous NiSO_4_ electrolyte that was very suitable for reversible deposition of Ni at a concentration of 1 M, and obtained a capacity of 298 mAh/g (at 200 mA/g) at an average voltage of 0.85 V (*vs.* Ni/Ni^2+^) for 2200 cycles [59]. This research has certainly opened the doors for exploring different multivalent ion batteries using a simple analogy of supercapacitors.

- ***Copper Hexacyanoferrate (CuHCF)***

Yi Cui and coworker’s [43] studies on intercalation of Ni^2+^ into CuHCF yielded a capacity around 20 mAh/g in the beginning, however it reduced to 10 mAh/g after 2000 cycles while testing at a rate of 5C. The reasons for decay in capacity and poor columbic efficiency were not conclusively determined in this work, however the authors speculated a possible swapping of metal ions of CuHCF frame work with the insertion ions. But the presence of multiple anodic and cathodic peaks represented possible insertion of guest ions into ferricyanide vacancies. They also confirmed the aiding role of ferricyanide vacancies and shielding effect of water molecules of CuHCF in the rapid kinetics of diffusion of guest ions, including Ni^2+^.

### 3.4. Mg–Ion Batteries

Despite the similarities of Mg and Li insertion into the hosts, most of the intercalation compounds that are suitable for electrode materials for Li-ion battery systems demonstrate a very poor chemical performance with Mg ions. Following are some of the current issues with Mg-ion batteries: (1) slow diffusion of Mg ions in the solids because of strong electrostatic interactions with the host structures; (2) growth of an insulating passivating surface film on the Mg-metal anode; (3) narrow electrical window of electrolytes used for the Mg-ion electrochemical activity; and (4) relatively low specific energy of the Mg-ion battery as compared to lithium [49]. Mg is a very active metal and is always covered with surface films, which usually consist of oxides, halides, hydroxides, and carbonates depending on the surrounding atmospheric conditions, solvents and gases. In addition, the passivation of Mg electrodes was observed to be quite rapid even with a small amount of moisture or water in the electrolytes [84]. However, the Mg electrodes are highly reversible only in Grignard reagents, where they can be dissolved and deposited using Grignard reagent based electrolytes, and in all other cases they tend to form passivating films with the compounds listed above. These passivating films prevent a continuous or spontaneous reactions between the active Mg and the electrolyte, and unfortunately they hamper the diffusion of Mg^2+^ unlike in Li-ion batteries, where the surface films formed on Li are usually Li-ion conducting in most of the commonly used electrolyte solutions [85]. In order to overcome these problems, use of acidic species in the electrolytes is suggested to help breakdown the passivating film and thereby to facilitate the interactions between Mg anodes and the electrolytes. In another approach, anodic polarization could also be implemented to cause the rupture of passivating films [85]. In aqueous electrolytes, the alloys of Mg could also be implemented as anodes for Mg-ion battery applications [20].

Recently, the electrodes of Chevrel phases (Mg*_x_*Mo_6_T_8_, T = S, Se) [41,86] and dichalcogenides (MoS_2_, WSe_2_) [87,88,89] were determined to have weak electrostatic interactions between Mg^2+^ and their anionic frameworks with moderate polarities. However, these anions with moderate polarities result in weak bond strength between the transition metals and surrounding anions leading to lower redox potentials of the transition metals, and thereby lower electrode potentials. Therefore, achieving both high voltages and efficient kinetics is also nontrivial for Mg-ion battery cathode materials. In addition, bivalency of Mg^2+^ imposes a higher energy penalty on desolvation at the electrode-electrolyte interface. There is plenty of research done on several cathode materials for Mg-ion batteries using non-aqueous electrolytes and their review can be found in [90], however using aqueous electrolytes more research is yet to be done. Despite many complexities and challenges, there are yet multiple electrode materials researchers have been able to investigate for their electrochemical characteristics. The subsections below focus on the electrode materials studied for Mg-ion battery applications using aqueous electrolytes and their electrochemical characteristics.

- **λ*-MnO_2_ /Spinel Mn_2_O_4_***

Among many materials that have been investigated, MnO_2_ has attracted much attention due to its tunnel structure, which can accommodate a large range of metal ions with different valences. MnO_2_ with various crystal structures, Hollandite, octahedral molecular sieve structure, and Birnessite (layered), Hollandite with large tunnels that are capable of accommodating various cations [91] demonstrated the highest possible capacity, but with a significant voltage hysteresis and slower Mg^2+^ diffusion kinetics in non-aqueous electrolytes [90]. Recently, it was experimentally [92] and theoretically [93] studied and showed to convert into amorphous phase upon insertion of Mg^2+^ in contrary to the regular intercalation phenomenon. Unfortunately, to confirm some of these observations in aqueous electrolytes, there is a lack of literature. Most of the aqueous electrolyte work was pursued on spinel structured compounds, which are discussed in detail below.

In 2014, Yuan *et al.* [20] reported the intercalation of Mg ions into λ–MnO_2_ that was prepared via acid leaching of LiMn_2_O_4_. This study focused on testing of λ–MnO_2_ in four different aqueous electrolytes ZnSO_4_, MgSO_4_, Mg(NO_3_)_2_ and MgCl_2_ and determined that λ–MnO_2_ was an ideal battery material for polyvalent cations (Mg^2+^, Zn^2+^) to intercalate/deintercalate. Mg^2+^ showed a better intercalation capability compared to Zn^2+^ as it has smaller size. Among all the electrolytes tested, λ–MnO_2_ performed the best in MgCl_2_ electrolyte showing a specific capacity of 400 mAh/g at a current density of 13.6 mA/g [20]. However, it reduced to 90.9, 93.2 and 155.6 mAh/g after the first 50 cycles in MgSO_4_, Mg(NO_3_)_2_ and MgCl_2_ electrolytes, respectively. Interestingly theses capacities remained constant up to 300 cycles. The λ–MnO_2_ electrode was also tested in MgCl_2_ electrolyte with different concentrations (0.5, 1, 3 M) and current densities (13.6, 27.2, 68, 136, 408 mA/g). It exhibited the largest discharge capacity at 0.5 M concentration and a poor performance at 3 M. The charge process and chlorine evolution reactions were observed to compete with each other, and limit the de-intercalation of Mg^2+^ from the λ–MnO_2_ electrode depending on the electrolyte concentration. The CV investigations revealed a preference for lower concentration of MgCl_2_ because of a large potential difference between the voltages required for chlorine evolution and the oxidation of the electrode, which facilitates the full deintercalation of Mg^2+^ before the evolution of chlorine starts.

In another study, Nupur and Munichandraiah [64] prepared MgMn_2_O_4_ in an aqueous Mg(NO_3_)_2_ electrolyte by electrochemically removing Li from LiMn_2_O_4_ using cyclic voltammetry. The average oxidation state of Mn was reduced to +3 with the additional charge of +2 of Mg in MgMn_2_O_4_. Later they tested these MgMn_2_O_4_ electrodes for charge-discharge cycling using aqueous Mg(NO_3_)_2_ and Mg(ClO_4_)_2_ electrolytes and obtained similar results. Galvanostatic charge–discharge cycling clearly indicated a good electrochemical reversibility of MgMn_2_O_4_ structures with λ-MnO_2_. The discharge cycles showed a maximum capacity of 42 mAh/g in the first cycle, which eventually reduced to ~35 mAh/g after 20 cycles. In a similar study to above, Kim *et al.* [66] also prepared a spinel Mn_2_O_4_ by de-lithiating LiMn_2_O_4_ in two stages, initially chemically and then following electrochemically in an aqueous Mg(NO_3_)_2_ solution. These authors have confirmed the formation of spinel Mn_2_O_4_ phase through crystallography and electron microscopy examination. During their electrochemical de-lithiation of spinel Mn_2_O_4_ phase, no cointercalation of water was confirmed in the electrode, as it would be usually expected from the small space available in the 3D spinel structures. The authors hypothesize that no-participation of water during de-lithiation should also be an indication for no interaction or participation of water in non-aqueous electrolytes during the electrochemical tests. Thus, it was tested with a fully delithiated Mn_2_O_4_ electrode assembled in a three electrode cell with Mg(TFSI)_2_ in dyglime or propylene carbonate electrolytes, and the cells showed a high voltage hysteresis with a lower degree of intercalation of Mg^2+^ into Mn_2_O_4_ compared to the aqueous electrolytes indicating considerable kinetic barriers in non-aqueous electrolytes.

- ***MnO_2_ Birnessite***

Nam *et al.* [65] did some very interesting studies to understand the electrostatic shielding behavior and structural stability provided by water molecules in a spinel structured Mn_3_O_4_ as well as converted Birnessite MnO_2_ electrodes. Their efforts initially focused on converting spinel Mn_3_O_4_ into Birnessite MnO_2_ structure by galvanostatically cycling it for 50 cycles in an aqueous 1 M MgSO_4_ electrolyte, which showed an increasing capacity up to 170 mAh/g because of co-intercalation of water into the electrode, and it resulted in an aqueous activated lamellar structured electrode turning from spinel Mn_3_O_4_ to Birnessite MnO_2_, which is quite contrasting to the studies conducted by Chunjoong Kim *et al.* [66] in spinel structures. Thus water activated electrode was then examined in non-aqueous electrolytes with and without addition of water. Their experiments revealed that the water in the host, *i.e.*, Birnessite MnO_2_, called crystal water, effectively screened the electrostatic interactions between Mg^2+^ and the host anions and thereby enhanced the electrochemical activity of Birnessite MnO_2_. Addition of water to the non-aqueous electrolyte solutions also allowed for hydration of Mg^2+^ at the surface of the electrodes and facilitated an easy intercalation of Mg^2+^ ions with lowered desolvation energy penalty at the electrode–electrolyte interface. The hydration of Mg^2+^ suppresses the coulombic repulsion between Mg^2+^ ions and the electrode surface and thereby assists in easy diffusion of Mg^2+^ into the electrode at the interface. In the case of pure non-aqueous electrolyte solution (Mg(ClO_4_)_2_ in acetonitrile), Birnessite MnO_2_ displayed a reversible capacity of only 56.8 mAh/g, however with increasing the water content it increased up to 230 mAh/g at a voltage of 2.8 V (*vs*. Mg/Mg^2+^) for a 10 M concentration of water in the electrolyte. It retained up to 80% of the initial capacity, *i.e.*, 183.2 mAh/g after 30 cycles at 100 mA/g. At different current densities of 200, 500, 1000, and 2000 mA/g the capacity retention was around 93.8%, 74.6%, 59.9% and 48.6% of initial capacity, respectively. These Birnessite MnO_2_ electrodes showed far better cycling stability in the water added non-aqueous electrolyte as opposed to pure non-aqueous electrolyte. These studies conveyed that a vast number of hydrated compounds that can effectively shield the electrostatic interaction between Mg^2+^ ions and the anionic framework of inorganic hosts may be considered as good candidates for Mg-ion battery cathodes.

- ***Nickel Hexacyanoferrate (NiHCF)***

Nickel hexacyanoferrate (NiHCF) was tested for Mg-ion batteries by Wang *et al.* [15]. They have used nanoparticles of NiHCF synthesized via co-precipitation method, and their electrochemical performance was evaluated in aqueous 1 M Mg(NO_3_)_2_ electrolyte (pH = 2) in a three electrode configuration with Ag/AgCl reference electrode, and a platinum counter electrode. However, for cyclability measurements, an activated carbon counter electrode was used. The initial capacity of NiHCF was ~52 mAh/g, but it decayed and stabilized at 65% of its initial capacity after 2000 cycles when it was tested at a rate of 5C, and the fading capacity was attributed to partial solubility of NiHCF in the electrolyte. The discharge processes of the electrodes were shown to be due to two phase reactions, which are however not known at this stage. The open framework crystal structure of NiHCF seemed to facilitate the high rate insertion of Mg^2+^ ions to produce exceptional cycle life and coulombic energy efficiencies. In addition, the size of nanoparticles, presence of hydrated ions in the crystal structure, and high ionic conductivity of NiHCF also helped in rapid kinetics and high rate capacities of the electrodes [15].

- ***V_2_O_5_ Xerogels***

V_2_O_5_ xerogel are very interesting materials for intercalation of ions because of their high surface area, short distances, and large interlayer spacing with water molecules. They resemble lamellar ordering with water molecules intercalated between the layers [94], and these water molecules could provide steric hindrance effect on the diffusion of multivalent ions. There is plethora of literature on intercalation characteristics of uni and multivalent ions into V_2_O_5_ xerogels (in non-aqueous electrolytes) available in the reference [94]. In 2010, for the first time, Stojkovic *et al.* [32] investigated the insertion of Mg^2+^ into V_2_O_5_ xerogels in aqueous Mg(NO_3_)_2_ electrolytes. In their research, they studied electrochemical characteristics of V_2_O_5_ xerogels and their composites with carbon and then compared with crystalline V_2_O_5_ [67]. Their charge-discharge cycling of V_2_O_5_ xerogels yielded a higher capacity of 50 mAh/g compared to the capacity of 30 mAh/g for the crystalline V_2_O_5_ [67]. The lower capacity of V_2_O_5_ was attributed to its insulating nature and slow diffusion kinetics of Mg^2+^ in V_2_O_5_. Recent work by Ceder *et al.* [95] also indicated slow diffusion kinetics in V_2_O_5_ electrodes, especially in the stable α-V_2_O_5_ phase, which is associated with a conversion of fully demagnesiated α-V_2_O_5_ into a coexisting two-phase structure with fully magnesiated δ-V_2_O_5_ and fully demagnesiated α-V_2_O_5_ phases during the discharge process. The experimental investigations by Aurbach *et al.* [96] also indicated possible multi-stage transformation during the intercalation with high resistivity of V_2_O_5_ phase. However, in experimental work, so far α-V_2_O_5_ is the only known electrode with reversibility of Mg^2+^ intercalation with slow diffusion kinetics. All these findings point to inferior quality of V_2_O_5_ compared to the xerogel structures of V_2_O_5_ for Mg battery electrode applications. Interestingly, addition of carbon seems to enhance the performance of V_2_O_5_ xerogels even more [32]. The composites of V_2_O_5_ xerogels with 30% carbon yielded even a higher capacity of 107 mAh/g, and this was attributed to more electrical conductivity of the composites with carbon addition, compared to the pure insulating V_2_O_5_ xerogels where carbon was added only 10% in their electrode preparation. Similarly, the CV data also exhibited higher peak currents for composite samples compared to the pure xerogels supporting the galvansotatic charge-discharge test data [32].

In another experimental and modeling study by Vujkovic *et al.* [33], the composites of V_2_O_5_ xerogels-Carbon showed more favorable intercalation for Mg^2+^ compared to the univalent Li^+^, K^+^ and Na^+^ in aqueous electrolytes. Interestingly, the capacity retained was also higher with insertion of Mg^2+^ into V_2_O_5_ xerogels-Carbon composites. The initial specific capacities obtained with insertion of Mg^2+^, Li^+^, K^+^ and Na^+^ were 102, 85, 85 and 87 mAh/g (at a current density of 1000 mA/g), respectively, and after 10 cycles, the retained capacity was observed to be 80, 42, 40 and 35 mAh/g, respectively. Through CV studies, it was determined that the interlayer site of V_2_O_5_ xerogel could be relatively more affordable for Mg^2+^ compared to the univalent ions, including Li^+^, although their sizes are comparable. The authors postulated that the insertion of Mg^2+^ in the interlayer sites of xerogels may not strain the crystal structure as much as the univalent ions do because of possible redistribution of intercalated Mg^2+^ between two non-equivalent sites, as well as redistribution of charge. They also conducted theoretical modeling studies using density functional theory (DFT) calculations to confirm the experimental observations. According to their DFT calculations, the Mg^2+^ interacts strongly with oxygen consisting V=O group (without collapsing the structure) compared to the monovalent ions and thereby helps in strengthening of the V–O(3) bonds and more stabilized xerogel structure. In case of univalent ions, the weaker interaction resulted in possible weaker bonding of V–O(3) bonds upon insertion of univalent ions and hence resulted in reduced number of vanadium ions in the crystal structure releasing them to dissolve in the electrolyte. Their modeling studies pointed toward application of smaller cation size to achieve better cyclic stability of the xerogel in aqueous electrolytes. Although, the ionic radii of Li^+^ (0.76 pm) and Mg^2+^ (0.72 pm) are very similar but higher charge of Mg^2+^ was attributed to help in achieving better cyclic stability. These studies certainly provided new insights into storage of multivalent ions in V_2_O_5_ xerogel–Carbon composites.

### 3.5. Other Ions (Ca, Ba, Cu, Sr, Nd, La, Y, Sm, Pb and Ce)

- ***Nickel Hexacyanoferrate (NiHCF)***

Similar to Mg-ion batteries, NiHCF was also investigated for reversible insertion of other alkaline earth divalent ions, including Ca^2+^, Sr^2+^ and Ba^2+^ by Wang *et al.* [15]. The aqueous electrolytes were prepared by dissolving appropriate metal nitrates in deionized water, and a 0.1 M concentration was used for Ba^2+^, and for all other cations the concentration was 1 M. For Ca^2+^, Sr^2+^ and Ba^2+^, the half charge reaction potentials with respect to the standard hydrogen electrode (SHE) were 0.59, 0.63 and 0.64 V, respectively. The NiHCF electrodes were observed to maintain a 53%, 64%, and 93% of their initial capacities after 2000 cycles for Ca^2+^, Sr^2+^, and Ba^2+^, respectively, at a rate of 5C. As explained earlier, the electrode particle size, ionic conductivity, presence of zeolitic water and open crystal structure of the active NiHCF were reasoned to help obtain the high rate capacities and high coulombic efficiencies [15]. These observations certainly indicate the versatile nature of NiHCF with high power capabilities for cathode applications in multivalent aqueous electrolyte intercalation batteries.

- ***Copper Hexacyanoferrate (CuHCF)***

As described in the section for Al-ion batteries, CuHCF was also investigated for insertion of many other multivalent ions, such as Cu^2+^, Co^2+^, Pb^2+^, Nd^3+^, La^3+^, Sm^3+^, Y^3+^, and Ce^3+^ by Yi Cui *et al.* [43], and their work showed a very good reversibility of intercalation of many of these trivalent metals without much decay in their capacity after several cycles, for example, Y^3+^ sustained a good cyclability of more than 2000 cycles. The effect of C rate was also observed to be not very substantial in terms of the decay of specific capacity for trivalent ions compared to the divalent ions. Intercalation of Y^3+^ showed a reduction of capacity from ~70 mAh/g to ~59 mAh/g while increasing the C rate from C/5 to a fifty fold increase, *i.e.*, 10C. The specific capacities of CuHCF with the insertion of Nd^3+^, La^3+^, Sm^3+^, Y^3+^, and Ce^3+^ was obtained around 39, 38, 36, 61 and 51 mAh/g, respectively. On the other hand, the divalent ions Cu^2+^, Co^2+^ and Pb^2+^ exhibited capacities around 35, 19 and 57 mAh/g, respectively. Their electrochemical data and crystal structure analysis clearly suggested the role of both ferricyanide vacancies and high degree screening of water molecules (from the crystal structure) in the intercalation of multivalent ions, making CuHCF a very unique electrode material for multivalent ion batteries.

## 4. Future Directions

Above literature on aqueous batteries certainly sheds light on current progress as well as future potentials and prospects of aqueous battery technologies, including their possible uses for practical applications. However, due attention should be paid to different technical challenges, such as side reactions, co-intercalation of proton into the electrode materials, change of pH of the electrolyte during charge-discharge processes, electrode dissolution, *etc.*, and, certainly, these will not only change the chemistry of aqueous batteries, but also strongly affect their capacity retention and cyclability, as mentioned earlier in the Section 2. The voltage windows for investigation of electrochemical characteristics should be selected depending on the crystal structures of the electrode materials as well as the chemistry of electrolytes employed to avoid dissociation of water as well as deep extraction of intercalating ions (similar to deep delithiation) from the electrode material. At the same time, future research should also focus on the following in order to enhance the capabilities of aqueous battery technologies:

- Discover and/or develop new cathode materials with high voltages and high capacities.
- Develop new cathode materials with good reversibility for intercalation and deintercalation of multivalent ions.
- Improve the performance of currently discovered cathode materials with means of alterations in the electrode chemistries (e.g., doping) and structures, electrolytes, *etc.*
- Develop mechanisms to increase over-potentials for hydrogen and oxygen evolution by tailoring the chemistry of water, electrolytes and possibly electrode structures.
- Develop mechanisms to reduce the solvation energies of multivalent ions.
- Extend the research to nanostructured and porous cathode materials.
- Investigate the composite electrode materials in combination with the electrode materials used for supercapacitors.
- Explore the mechanisms and opportunities to enhance the interfacial capacitance at cathode/electrolyte interfaces.
- Reduce the polarization issues.

## Figures and Tables

**Table 1:** Comparison of standard electrode potentials and theoretical capacities of univalent and multivalent anodes.

Ion	Standard Electrode Potential (V)	Theoretical Capacity	
		Specific Capacity (mAh/g)	Volumetric Capacity (mAh/cm^3^)
Li^+^	−3.05	3829	2044
Na^+^	−2.71	1165	1128
Mg^2+^	−2.36	2234	3882
Ca^2+^	−2.87	1337	2073
Ni^2+^	−0.257	913	8133
Zn^2+^	−0.76	820	5854
Al^3+^	−1.66	2980	8046
